# Rapid Increase of Scrub Typhus: An Epidemiology and Spatial-Temporal Cluster Analysis in Guangzhou City, Southern China, 2006–2012

**DOI:** 10.1371/journal.pone.0101976

**Published:** 2014-07-09

**Authors:** Yuehong Wei, Yong Huang, Lei Luo, Xincai Xiao, Lan Liu, Zhicong Yang

**Affiliations:** Guangzhou Center for Disease Control and Prevention, Guangdong Province, Guangzhou, China; University of Malaya, Malaysia

## Abstract

**Background:**

Scrub typhus has been increasingly reported in Southern China, and public health authorities are concerned about its increased incidence. Additionally, little evidence is available on the epidemiology of scrub typhus in Southern China. This study aims to analyze the epidemiological and geographic features of ST in Guangzhou City, Southern China, to guide the future prevention efforts.

**Methods:**

Scrub typhus surveillance data in Guangzhou City during 2006–2012 were obtained from the Chinese National Communicable Disease Surveillance Network. We first conducted a descriptive analysis to analyze the epidemiological features of scrub typhus. Then we used space-time scan statistic based on a discrete Poisson model to detect and evaluate high-risk spatial-temporal clusters of scrub typhus.

**Results:**

There were 4,001 cases of scrub typhus in Guangzhou City during the study period. The incidence of scrub typhus increased from 3.29 per 100,000 in 2006 to 9.85 per 100,000 in 2012. A summer peak was observed in June and July with a second peak in September and October except year 2009 and 2011. The majority of the cases (71.4%) were among persons aged ≥40 years, and female incidence was higher than male incidence in persons ≥50 years. In the space-time analysis, high-risk clusters were concentrated in rural areas in Guangzhou City. Over the past 7 years, Haizhu District, an urban area, was found to be a high-risk cluster for the first time in 2012.

**Conclusion:**

The resurgence of scrub typhus epidemics in Guangzhou population in 2012 necessitates more effective measures for minimizing future epidemics. Consideration of high-risk population and historical spatial-temporal clusters may help prevent scrub typhus. The risk of scrub typhus in urban areas should not be neglected and needs more attention from public health authorities.

## Introduction

Scrub typhus (ST), or tsutsugamushi disease, is a rickettsial disease, which is highly endemic in the “tsutsugamushi triangle” extending from Afghanistan to China, Korea, the islands of the western Pacific and Indian Oceans, and northern Australia [1], [2]. The record of apparent ST was first described in China in AD 313[3]. The etiologic agent, *Orientia tsutsugamushi*, is transmitted to humans through the bite of infected trombiculid mite larvae of the genus *Leptotrombidium*. Clinical presentations of ST could be fatal with increased age and greater bacterial load, and the untreated case-fatality rate is approximately 7% [4]. Recently, the resurgence of ST has been documented in India, South Korea, northern China, and Taiwan [5]–[10]. More than one billion persons are at risk for scrub typhus, and about one million new cases occur every year [11].

In China, ST was first found in Guangzhou City in 1948. Since then, the disease has been increasingly reported in many other parts of China, particularly in southern China [12], [13]. Given the large populations of China, the numbers potentially exposed are enormous. Guangzhou City is the only city listing ST as a notified infectious disease in mainland China [12], with ST incidence increasing sharply in recent years. In 2006, the number of ST cases in Guangzhou City was 321. The number almost doubled in 2011 (653 cases), was four-fold in 2012 (1256 cases). ST is also expected to increase in future due to the increasing outdoor activities of urban inhabitants, the aging of population, improved surveillance and diagnostic methods, climate change, greater density of rodent and chigger mites and the lack of health resources [14]–[17].

Until now, no population-wide information on ST has been available in southern China, and the hotspots or clusters of ST in Guangzhou remained unclear. A better understanding of epidemiological and geographic features of ST may help identify the population and areas at high risk, and might provide useful insights into surveillance, local epidemic control and resource allocation. Therefore, we conducted this study to analyze the epidemiological features of ST, and identify the spatial-temporal high risk clusters in Guangzhou City during 2006–2012 for further public health interventions.

## Materials and Methods

### Ethical statement

No work with human subjects was directly involved in our study. ST data were extracted from Chinese National Communicable Disease Surveillance Network. All patient records were anonymized and de-identified prior to analysis. Permission to conduct this study was approved by Guangzhou Center for Disease Control and Prevention Ethics Committee and Guangzhou Health Bureau.

### Study site

**Figure 1:**
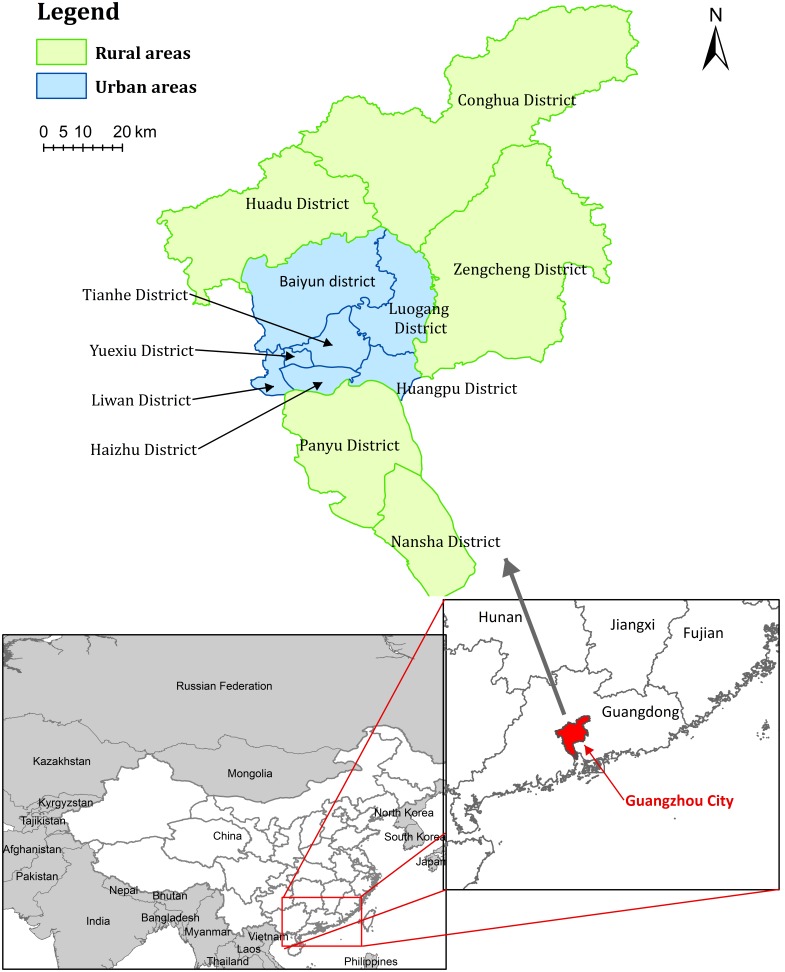
Geographic location of Guangzhou city, Guangdong province, China.

Guangzhou, the capital city of Guangdong Province and the biggest city in Southern China, has a population density of 1,720 persons per km^2^ (in 2012: population = 12.75 million; land size = 7434.4 km^2^). It is situated in the northern hemisphere with an annual average relative humidity of 75% and a temperature of 22°C [18]. Guangzhou has twelve districts, and is divided into rural and urban areas according to the land using and economic development (Figure 1).

### Surveillance of scrub typhus

The National Communicable Disease Surveillance Network, now covering a catchment of approximately 13 million inhabitants in Guangzhou City, has provided a powerful tool for guiding and evaluating ST prevention strategies. Data of daily reported ST cases in Guangzhou City from January 1, 2006 to December 31, 2012 were obtained from Chinese National Communicable Disease Surveillance Network. Every medical institution was required to report ST cases daily via the web-based surveillance system with unified format, including the information about sex, age, residential address, occupation and date of symptom onset.

The criteria for diagnosis of ST were provided in a guidebook published by the Chinese Center for Disease Control and Prevention[19]. Patients with the following items are defined as clinically diagnosed cases: (i) Field exposure history 1–3 weeks prior to symptoms; (ii) Fever with skin rash orlymphadenopathy; (iii) Typical eschars or ulcers. Samples from clinically diagnosed cases are then detected at Guangzhou Center for Disease Control and Prevention laboratory with indirect immunofluorescence antibody assay (IFA) using mixed Karp, Gilliam and Kato strains of *O. tsutsugamushi* as diagnostic antigen (IgM/IgG titer ≥4-fold increase in paired serum) and nested polymerase chain reaction (PCR) test targeting 56-kDa gene of *O. tsutsugamushi* for laboratory confirmation [19]. Our analyses were based on the laboratory confirmed cases.

### Descriptive epidemiology analysis

The epidemiological characteristics including demographic, temporal and spatial distribution of ST cases from 2006 to 2012 in Guangzhou City were analyzed according to surveillance data. Incidence rates were expressed as the number of new cases per 100,000 population in a given time period. The populations for years 2006–2012 provided by Guangzhou Statistical Bureau were used as denominators.

### Space-time cluster analysis

High risk clusters of ST were detected with a retrospective space-time scan statistic based on a discrete Poisson model, using SaTScan software (version 9.1.1). The space-time scan statistic is defined by a cylindrical window with a circular geographical base which is centered on the centroids of areas, and with height corresponding to time [20]. The null hypothesis assumed that the relative risk (RR) of the incidence was the same within the window as compared with outside [21]. The base and the height of the windows are dynamic in order to detect possible sub-clusters. The difference of the incidence inside and outside the windows was evaluated by the Log Likelihood Ratio (*LLR*):
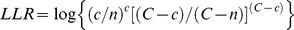
Where C denotes the total number of cases; c is the number of observed cases inside the window; n is the number of expected cases inside the window. The window with largest *LLR* value is defined as the primary cluster; other windows with statistically significant *LLR* values are defined as secondary clusters. Statistical significance was evaluated in a Monte Carlo simulation method, and a window with a *P* value less than 0.05 was identified as a statistically significant cluster [22].

In this study, we performed the space-time scan statistic annually to observe the cluster changes and adjust for the temporal trend during the study period [23]. The maximum radius of circular base was set at 50% of the total population at risk and the maximum height of the cylinder was set at 50% of the total study period. The number of Monte Carlo replications was set to 999 and the significance level was set at 0.05.

## Results

### Descriptive epidemiology

**Figure 2:**
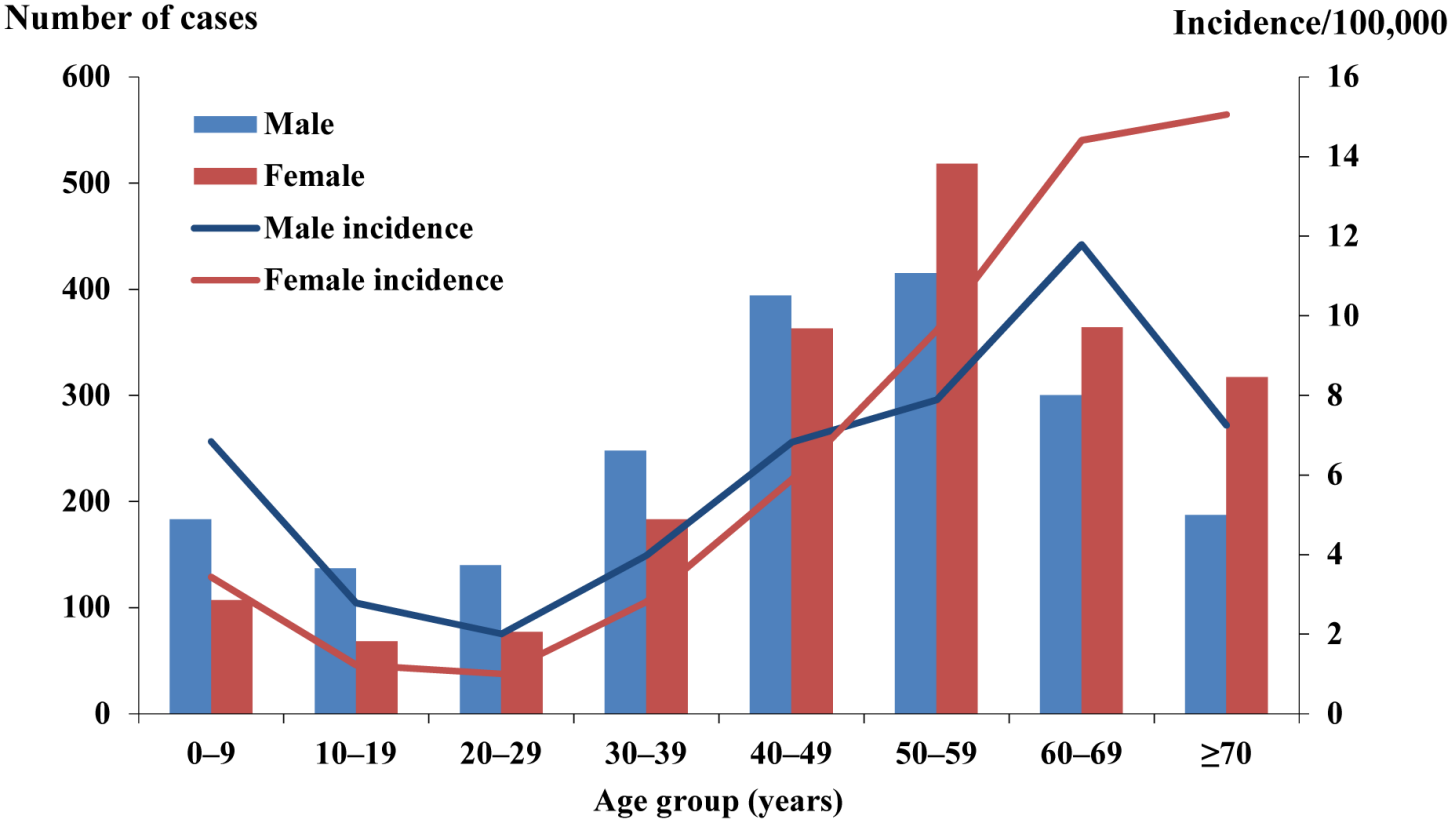
Age and sex distribution of reported scrub typhus cases in Guangzhou city, China, 2006–2012.

**Figure 3:**
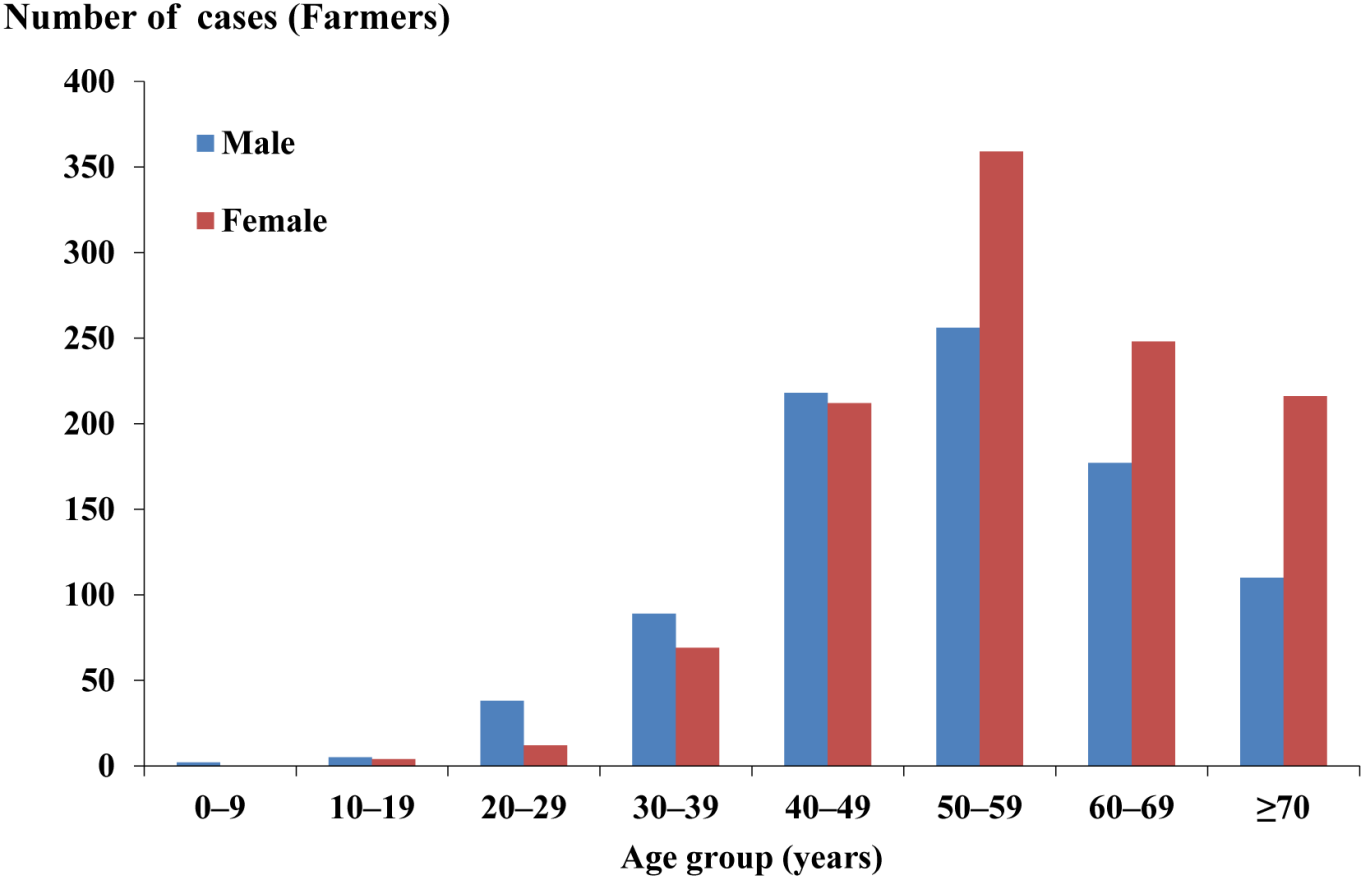
Age and sex distribution of reported scrub typhus cases (farmers) in Guangzhou city, China, 2006–2012.

A total of 4,001 ST cases were reported in Guangzhou city during the 7-year study period. Of these, 50.1% were male and 49.9% were female. The female incidence rates were higher than male incidence rates in persons 50–59, 60–69, and ≥70 years (Figure 2). Regarding occupation, 50.4% of ST cases were farmers, followed by the unemployed and the retired (14.4%), workers (9.5%) and students (5.8%). For farmers, the percentages of female cases were higher than those of males in persons 50–59, 60–69, and ≥70 years (Figure 3).

**Figure 4:**
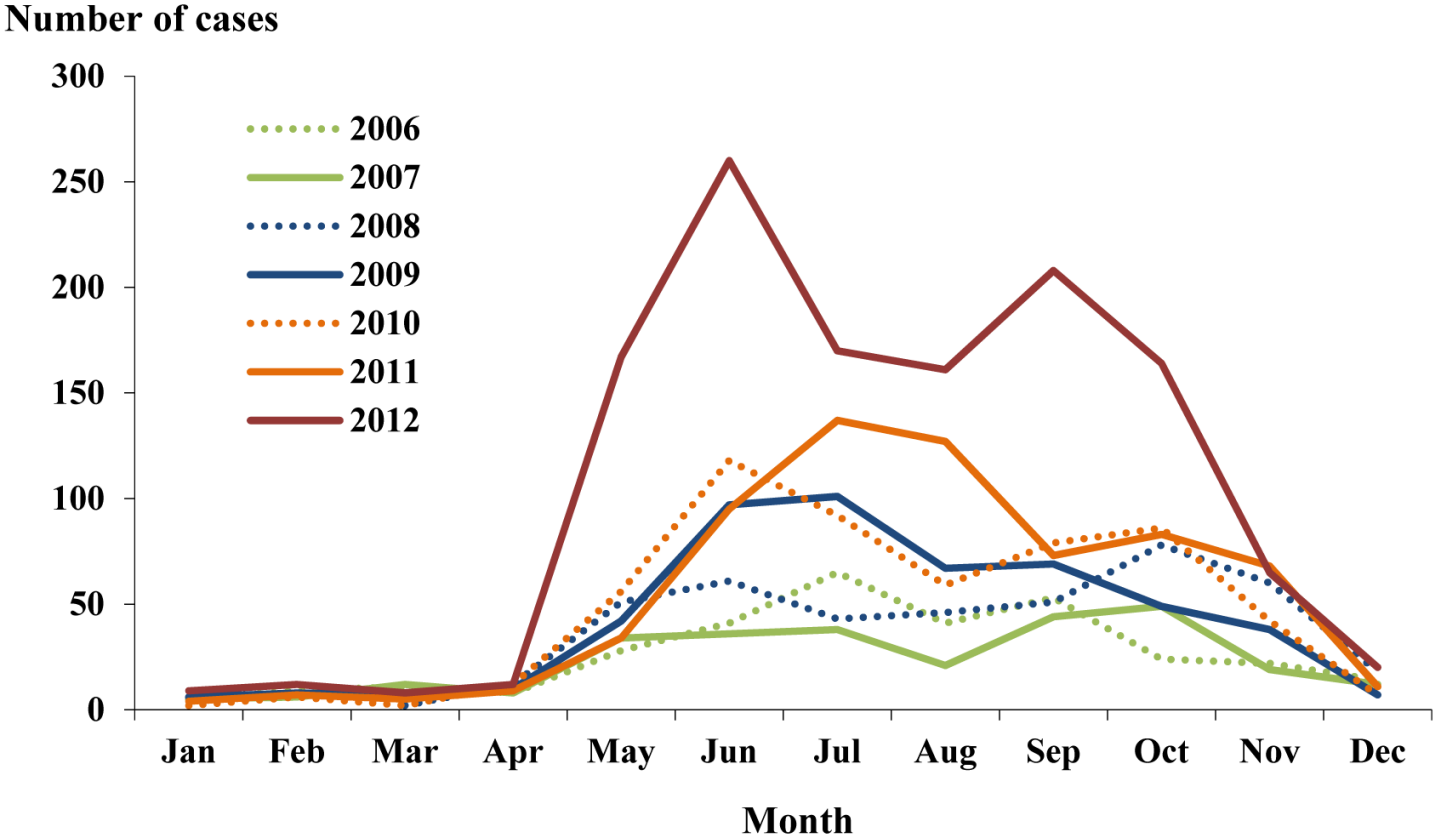
Seasonal distribution of reported scrub typhus cases in Guangzhou city, China, 2006–2012.

The temporal trend and seasonal distribution of ST cases was shown in Table 1 and Figure 4, respectively. The overall annual incidence rates increased from 3.29 per 100,000 in 2006 to 9.85 per 100,000 in 2012 (Cochran Armitage trend test, p<0.001). During the 7-year study period, a summer peak was observed in June and July with a second peak in September and October except year 2009 and 2011.

**Table 1:** Demographic characteristics of reported scrub typhus cases in Guangzhou city, China, 2006–2012.

Parameters	Cases	Incidenceper 100,000	Proportion	Female% vs.male%
**Total**	4,001	5.26	100.0	50∶50
**Year**				
2006	321	3.29	8.0	49∶51
2007	284	2.83	7.1	50∶50
2008	425	4.17	10.6	50∶50
2009	501	4.88	12.5	47∶53
2010	561	5.43	14.0	48∶52
2011	653	5.14	16.3	50∶50
2012	1,256	9.85	31.4	52∶48
**Age**				
0–9	290	5.00	7.2	37∶63
10–19	205	1.96	5.1	33∶67
20–29	217	1.48	5.4	35∶65
30–39	431	3.38	10.8	42∶58
40–49	757	6.33	18.9	48∶52
50–59	933	8.76	23.3	56∶44
60–69	664	13.10	16.6	55∶45
≥70	504	10.75	12.6	63∶37
**Gender**				
Male	2,004	5.42	50.1	-
Female	1,997	5.11	49.9	-
Sex ratio	1.00	1.06	-	-
**Occupation**				
Farmers	2,015	-	50.4	57∶43
The unemployed and the retired	657	-	14.4	57∶43
Workers	383	-	9.5	39∶61
Students	232	-	5.8	32∶68
Preschoolers	185	-	4.6	37∶63
Others	529	-	13.2	44∶58
**Geographic area**				
Rural	2,675	9.59	65.0	51∶49
Urban	1,440	2.99	35.0	48∶52

**Figure 5:**
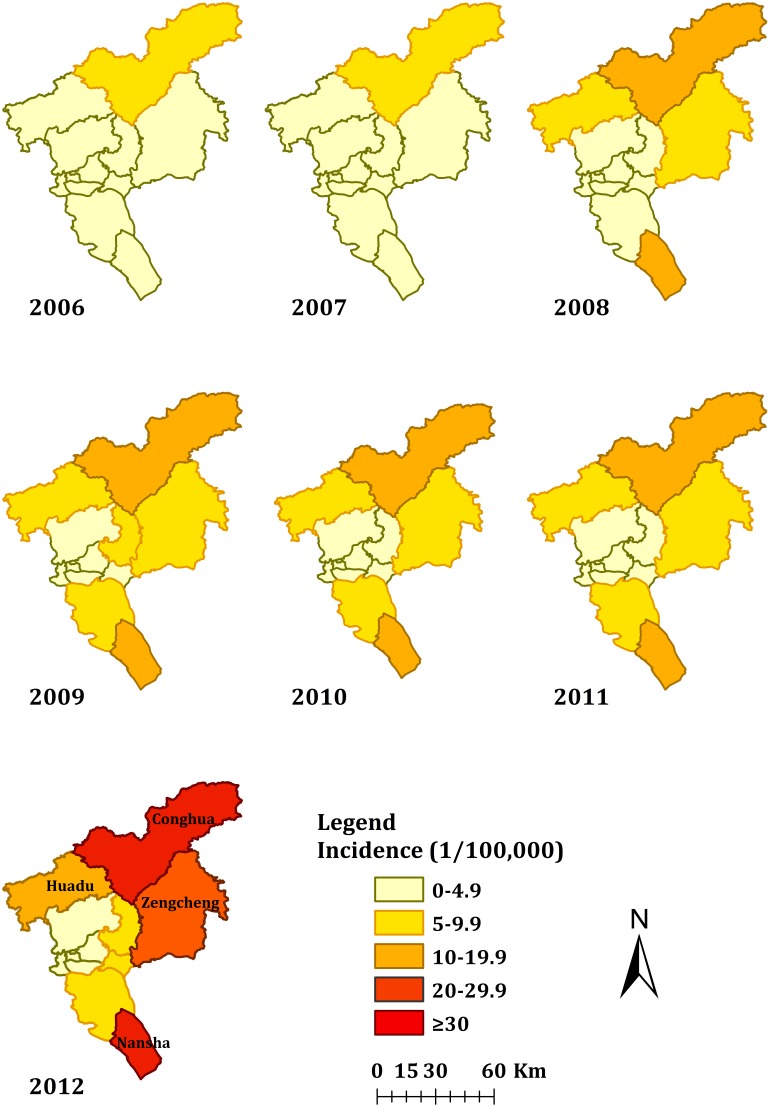
Spatial distribution of reported scrub typhus cases in Guangzhou city, China, 2006–2012.

The main endemic areas of ST were located in rural areas, which accounted for 65.0% of the reported cases (Table 1). The spatial distribution of ST per year was shown in Figure 5, which indicated that the annual incidence per district had sharply increased from 2006 to 2012. Conghua, Zengcheng, Nansha and Huadu were the top four regions in incidence rate.

### Spatial-temporal clusters of scrub typhus

**Figure 6:**
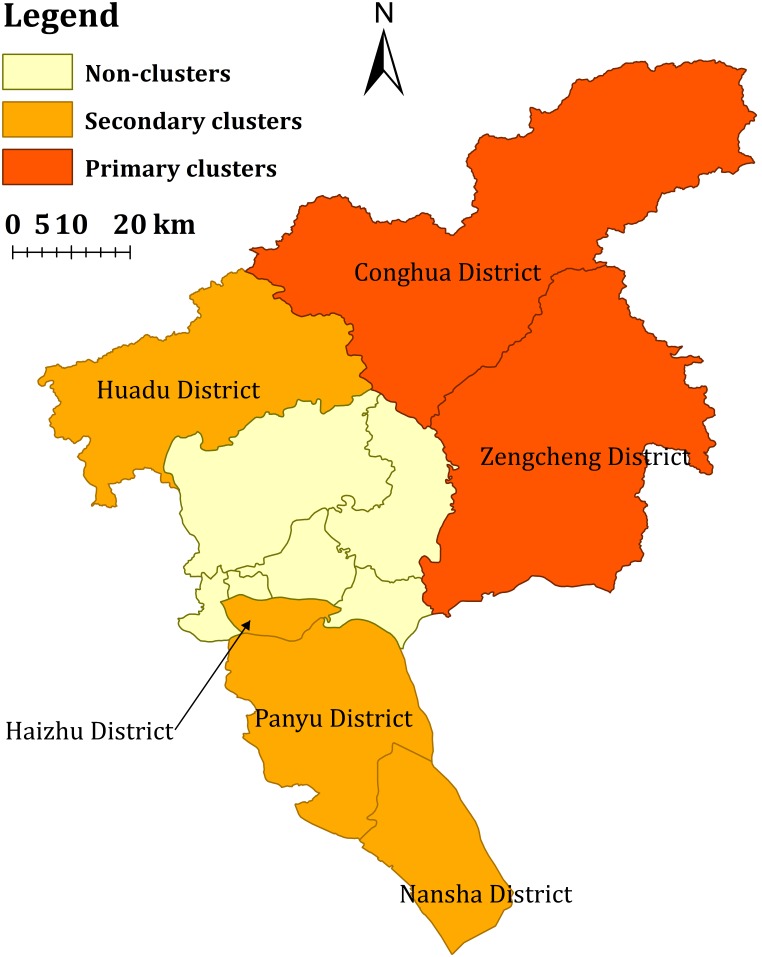
Space-time clusters of reported scrub typhus cases in Guangzhou city, China, 2006–2012.

The primary clusters for high incidence of ST were found at Conghua and Zengcheng Districts from May to November. Secondary clusters detected for high incidence of ST were located in Nansha, Panyu and Huadu District mainly from May to September. Over the past 7 years, Haizhu District, an urban area, was found to be a new cluster for the first time in 2012. (Table 2 and Figure 6).

**Table 2:** Spatial-temporal clusters of scrub typhus in Guangzhou city, China, 2006–2012.

Year	Cluster type*	Time	Location	No. Obs	No. Exp	RR	P
**2006**	1	5/22–10/22	Conghua, Zengcheng	52	18	3.3	<0.001
	2	7/3–7/30	Nansha, Panyu	19	5	4.1	<0.001
	3	6/5–9/24	Huadu	23	7	3.5	<0.001
**2007**	1	5/22–11/19	Conghua, Zengcheng	74	18	5.1	<0.001
	2	5/8–11/5	Nansha, Panyu	49	24	2.3	0.002
	3	5/8–10/22	Huadu	28	9	3.2	<0.001
**2008**	1	5/22–11/19	Conghua, Zengcheng,	124	28	6.0	<0.001
	2	5/8–11/5	Nansha, Panyu	90	35	3.0	<0.001
	3	5/8–7/16	Huadu	18	6	3.2	0.036
**2009**	1	5/22–11/5	Conghua, Zengcheng	165	32	7.1	<0.001
	2	5/22–9/10	Nansha, Panyu	82	26	3.6	<0.001
	3	5/22–9/10	Huadu	32	11	3.0	<0.001
**2010**	1	5/22–11/19	Conghua, Zengcheng	161	36	5.8	<0.001
	2	5/22–7/16	Nansha, Panyu	91	15	7.4	<0.001
		5/22–11/19	Huadu	50	19	2.9	<0.001
**2011**	1	5/22–11/19	Conghua, Zengcheng,	234	42	8.2	<0.001
	2	6/5–8/13	Nansha, Panyu	90	20	5.1	<0.001
	3	7/3–8/27	Huadu	34	8	4.8	<0.001
**2012**	1	5/22–11/5	Conghua, Zengcheng	408	75	7.6	<0.001
	2	5/8–6/18	Nansha, Panyu	105	23	4.9	<0.001
	3	5/22–10/8	Huadu	123	36	3.7	<0.001
	4	5/8–6/18	Haizhu	38	18	2.2	0.025

*1: Primary cluster; 2–4: Secondary cluster; No. Obs: number of observed cases; No. Exp: number of expected cases; RR: relative risk for the cluster compared with the rest of the city.

## Discussion

We observed that the number of ST cases was prominent in farmers (50.4%), which was similar to the findings in other Asian areas [8], [9]. Furthermore, most infected farmers were elderly women≥50 years (Figure 3). These findings might be explained by the unbalanced development between rural and urban areas, which results in the problem of left-behind children, the elderly and women. Most young, male people of rural families work in the urban areas to alleviate their economic burden, and the elderly and women are left to do farm work. Our results also found that male incidence rates were greater than female incidence rates in the age group 0–9, 20–29 30–39, and 40–49 years (All p<0.05), which reflect the conventional working behavior[24]. This pattern was supported by the male-female ratio in students (68∶32) and workers (61∶39). Interestingly, we observed an increasing number (proportion) of the unemployed and the retired from 44 (13.7%) in 2006 to 254 (20.2%) in 2012, and no studies has been reported to explain this phenomenon. Therefore the mechanism and risk factor of infection among unemployed remains an open question.

During the 7-year study period, ST incidence in Guangzhou city increased from 3.3 per 100,000 in 2006 to 9.9 per 100,000 in 2012. In particular, there was obvious increase in the number of reported cases in the years of 2012, which could be explained by a true higher incidence, as well as improved surveillance. The seasonality of ST detected in Guangzhou City showed two seasonal peaks in summer and autumn. Generally, seasonal distribution of scrub typhus is associated with the local period of trombiculid mite larval appearance. *L. deliense*, a common mite in Guangzhou City, appears from May to October with a single peak occurrence between June and August in the summer[25], [26]. However, the second peak in the number of cases occurred in October, which is inconsistent with the occurrence of *L. deliense*. This finding might suggest the co-circulation of two species of trombiculid mites. Unfortunately, there are no reports on the species and the natural foci of trombiculid mite in Guangzhou city since 1952. In addition, the long-time field activities of farmers, abundant rainfall in summer-autumn season and high density of rodents may increase the chances of exposure to the infected mites.

ST is widespread in rural areas of Guangzhou City, which is similar to the patterns of geographic distribution in Southeast Asia [5], [8], [27]–[29]. However, spatial variation in incidence might also reflect the regional surveillance and diagnosis. The high endemicity in rural areas has been associated with people in rural areas who are exposed to environmental factors such as crops, bushes, animal pets and rodents [30], [31]. The significant increase in incidence in these regions is particularly concerning and requires an investigation. Additional studies including cases and non-cases for comparison are needed to clarify the driving factors.

Surprisingly, Haizhu District, an urban area, was found to be a new cluster in 2012, although most clusters of ST were concentrated in rural Guangzhou. In 2012, Guangzhou CDC confirmed an atypical outbreak in a park in Haizhu District. Activity history in park, sitting on the lawn and sitting near the rat holes were associated with the risk of ST (data unpublished). This indicated that park is likely providing a suitable habitat for rodents and chiggers. Due to the improvement of living standards and operation of Guangzhou Metro, people have more chances to go out to the park for outdoor activities, which might contribute to a higher chance of infection [32]. The risk of ST in these areas should not be neglected and needs more attention from public health authorities.

Our study has some limitations. Firstly, the surveillance data were obtained from a passive reporting system, which indicated that some cases of ST might go underreported because of the nonspecific clinical symptoms [5], [33]. Secondly, we failed to isolate *O. tsutsugamushi* from patients by inoculating laboratory mice or cell culture and identify the strain of *O. tsutsugamushi*. In southern China, Karp types were the key genotypes of summer-type scrub typhus [12]. Although Gilliam types were identified as the key serotypes of autumn-winter type scrub typhus in many areas of northern China, the genotyping results acquired in Shandong province, northern China showed that Kawasaki types were the key genotypes of this type of scrub typhus [5], [7], [33]. Genetic characterization of *O. tsutsugamushi* should be considered in future study.

Despite these limitations, to the best of our knowledge this is the first study in Southern China using population-wide monitoring data to provide useful information on the epidemiological features of ST, and identified spatial-temporal high risk clusters at the district level over the last 7 years. This can help public health authorities to target vulnerable populations and augment control measures in the identified areas of high ST prevalence. In addition, this study represents an important first step towards understanding potential underlying causes of ST, which may give valuable clues for further studies.

In conclusion, the resurgence of scrub typhus epidemics in Guangzhou population in 2012 heightens the importance of more effective measures for minimizing future epidemics. A comprehensive preventive strategy including public health education and promotion, rodent control and surveillance should be implemented in the rural areas of Guangzhou in summer/autumn and in the urban areas of Guangzhou in summer. Further studies are needed to explore the role of the environmental factors (e.g. climate and landscape) and social factors (e.g. agricultural activities and human movements), abundance of chiggers, risk factors of personal behaviors, rodent control measures in driving the spatial-temporal distribution identified in this study.
